# Noninvasive Monitoring of Liver Disease Regression after Hepatitis C Eradication Using Gadoxetic Acid-Enhanced MRI

**DOI:** 10.1155/2018/8489709

**Published:** 2018-07-12

**Authors:** Lukas Haider, Mattias Mandorfer, Zeynep Güngören, Thomas Reiberger, Nina Bastati, Jacqueline C. Hodge, David Chromy, Michael Trauner, Christian Herold, Markus Peck-Radosavljevic, Ahmed Ba-Ssalamah

**Affiliations:** ^1^Department of Biomedical Imaging and Image Guided Therapy, Medical University of Vienna, Waehringer Gürtel 18‐20, 1090 Vienna, Austria; ^2^Division of Gastroenterology and Hepatology, Department of Internal Medicine III, Medical University of Vienna, Waehringer Gürtel 18‐20, 1090 Vienna, Austria; ^3^Radiology Department, Istanbul Medipol University, TEM Avrupa Otoyolu Göztepe Çıkışı No. 1 Bağcılar, 34214 İstanbul, Turkey; ^4^Department of Gastroenterology, Hepatology, Endocrinology and Nephrology, Klinikum Klagenfurt am Wörthersee, Feschnigstrasse 11, 9020 Klagenfurt, Austria

## Abstract

We evaluated changes in relative liver enhancement (RLE) obtained by gadoxetic acid-enhanced MRI (GA-MRI) in the hepatobiliary phase and changes in splenic volume (SV) after hepatitis C virus (HCV) eradication as well as their predictive value for the development of (further) hepatic decompensation during follow-up. This retrospective study comprised 31 consecutive patients with HCV-induced advanced chronic liver disease who underwent GA-MRI before and after successful interferon-free treatment, as well as a cohort of 14 untreated chronic HCV-patients with paired GA-MRI. RLE increased by 66% (20%–94%; *P* < 0.001) from pre- to posttreatment, while SV decreased by −16% (−28% to −8%; *P* < 0.001). However, SV increased in 16% (5/31) of patients, the identical subjects who showed a decrease in RLE (GA-MRI-nonresponse). We observed an inverse correlation between the changes in RLE and SV (*ρ*=−0.608; *P* < 0.001). In the untreated patients, there was a decrease in RLE by −11% (−25% to −3%; *P*=0.019) and an increase in SV by 23% (7%–43%; *P*=0.004) (both *P* < 0.001 versus treated patients). Interestingly, GA-MRI-nonresponse was associated with a substantially increased risk of (further) hepatic decompensation 2 years after the end of treatment: 80% versus 8%; *P* < 0.001. GA-MRI might distinguish between individuals at low and high risk of (further) hepatic decompensation (GA-MRI-nonresponse) after HCV eradication. This could allow for individualized surveillance strategies.

## 1. Introduction

Chronic hepatitis C virus (HCV) infection affects about 80 million people worldwide [1]. Ongoing hepatic inflammation may lead to liver fibrosis, cirrhosis, and ultimately portal hypertension, which may be complicated by ascites, variceal bleeding, and hepatic encephalopathy. Moreover, patients are at considerable risk for the development of hepatocellular carcinoma (HCC) [2].

The use of interferon- (IFN-) based therapies in patients with advanced liver disease was limited due to adverse events as well as its modest efficacy [3, 4]. In contrast, novel IFN-free regimens are highly effective and generally well tolerated [5–8]. Touting rates of sustained virologic response (SVR), which defines the cure of chronic hepatitis C (CHC), exceeding 95% [5–7], the focus of attention has now shifted to the regression of HCV-induced liver fibrosis, cirrhosis, and portal hypertension after treatment [9].

Monitoring patients who have achieved an SVR remains a major challenge in the post-HCV era [10]. In the absence of well-established biomarkers for risk stratification, patients who have achieved an SVR but had advanced liver fibrosis or cirrhosis before treatment should undergo upper GI endoscopy to screen for varices in 1- to 3-year intervals and have ultrasound (US) surveillance for HCC every 6 months [10, 11]. However, the US has many limitations, resulting in either false-positive or false-negative findings, and thus, overtreatment or undertreatment of a substantial proportion of patients [12].

Gadoxetic acid-enhanced MRI (GA-MRI) is considered more sensitive for diagnosis of HCC [12]. Based upon its unique pharmacokinetic properties, GA-MRI simultaneously gives morphologic and functional information about the hepatobiliary system in patients with diffuse liver diseases. The RLE measured 20 minutes after GA administration, that is, in the hepatobiliary phase (HBP), has been recently shown to correlate with liver function [13]. RLE facilitates selecting patients for major liver resection likely to develop liver failure postoperatively [14]. Likewise, the RLE has been shown to be a predictor of graft survival after liver transplantation [15]. RLE has been recently shown to correlate with hepatic inflammation and fibrosis in nonalcoholic steatohepatitis (NASH) [16]. Thus, we hypothesized that the RLE might identify patients who have persistent hepatic inflammation and fibrosis, as well as portal hypertension despite HCV eradication.

We therefore analyzed CHC patients with advanced chronic liver disease who achieved an SVR on IFN-free regimens and underwent GA-MRI both before and after IFN-free treatment to evaluate its potential use as a noninvasive tool for distinguishing between patients at low and high risk of (further) hepatic decompensation during follow-up. We used the change in relative liver enhancement (RLE) derived from GA-MRI in the HBP and the change in splenic volume (SV) as potential indicators of regression of hepatic fibrosis and inflammation [16] and improvement of portal hypertension, respectively [17, 18]. The changes in RLE and SV observed after HCV eradication were compared to the course of untreated chronic HCV infection assessed by paired GA-MRI.

## 2. Materials and Methods

### 2.1. Study Design and Population

**Figure 1:**
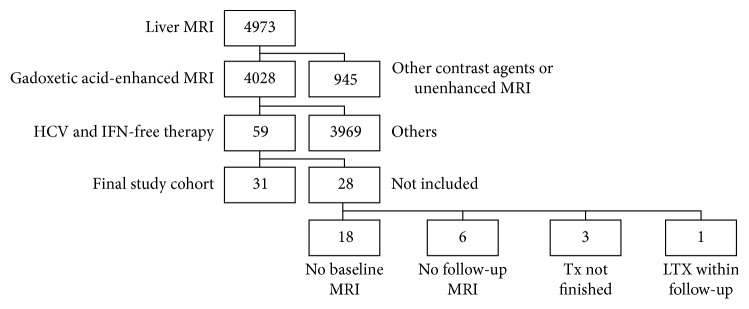
Between June 2011 and April 2016, 4973 patients underwent a standardized 3.0 Tesla MRI of the liver. Fifty-nine patients with chronic hepatitis C virus (HCV) infection who were successfully treated with interferon- (IFN-) free regimens underwent a GA-MRI of the liver in the course of hepatocellular carcinoma surveillance. Paired measurements were available in 31 patients (final study cohort); Tx: treatment; LTX: liver transplantation.

The Institutional Review Board of our hospital approved the data collection method and analysis and waived the necessity for informed consent for this retrospective study. A query of our institutional database yielded 4973 patients who underwent a standardized 3.0 Tesla MRI (3.0 T-MRI) of the liver between June 2011 and April 2016. Our inclusion criteria were met by 31 patients with CHC in whom GA-MRI liver was performed in the course of HCC surveillance both before and after IFN-free treatment. All of these patients had a SVR. The exclusion criteria are depicted in Figure 1. Patient characteristics are shown in Table 1.

In addition, our study comprised a cohort of 14 untreated chronic HCV patients with paired GA-MRI.

### 2.2. MRI Protocol

All MRI examinations were performed with a 3.0 T-MRI unit (TrioTrim, Siemens, Erlangen, Germany). The detailed MRI protocol including the examination parameters is shown in Table 2.

### 2.3. Clinical and Laboratory Parameters

Epidemiological characteristics were assessed from patients' medical history. Pretreatment model for end-stage liver disease (MELD) and Child-Pugh (CP) score was calculated based on laboratory parameters and patients' medical histories. Laboratory parameters were assessed using standard laboratory methods and recorded at the time of the 1st (before antiviral therapy) and the 2nd (after antiviral therapy) GA-MRI.

HCV genotype was determined using the VERSANT HCV Genotype 2.0 Assay Line Probe Assay (LiPA) (Siemens Healthcare Diagnostics, Tarrytown, NY, USA). HCV-RNA was assessed using the Abbott RealTime HCV assay (Abbott Molecular, Des Plaines, IL, USA) with a lower limit of quantification and detection of 12 IU  mL^−1^. SVR was defined by undetectable HCV-RNA 12 weeks after the end of antiviral therapy.

### 2.4. HCV Therapy

Patients were treated with sofosbuvir (SOF) in combination with simeprevir (SMV), daclatasvir (DCV), or ledipasvir (LDV), or the 3D regimen. SOF (Sovaldi (Gilead, Cambridge, UK) 400 mg once daily), SMV (Olysio (Janssen, Beerse, Belgium) 150 mg once daily), DCV (Daklinza (Bristol-Myers Squibb, Uxbridge, UK) 60 mg once daily), SOF/LDV (Harvoni (Gilead, Cambridge, UK) 400 mg/ 90 mg once daily), and the 3D regimen (Viekirax (AbbVie, Maidenhead, UK) 12.5 mg ombitasvir, 75 mg paritaprevir, and 50 mg ritonavir once daily plus Exviera (AbbVie, Maidenhead, UK) 250 mg dasabuvir twice daily) were either prescription drugs or provided by pharmaceutical companies. Treatment durations ranged from 12 to 24 weeks.

### 2.5. Image Analysis

The measurements of signal intensity (SI) were performed on a commercially available picture archiving and communication system workstation (IMPAX EE R20 XV SU3, AGFA Healthcare, Mortsel, Belgium) by three readers in consensus: a radiologist with more than 20 years of experience in abdominal MR imaging (Ba-Ssalamah) and two radiologists in the 2nd-3rd year of training (Haider and Güngören). All observers were blinded to patients' clinical histories and laboratory data, as well as the time point. The signal intensity of the liver parenchyma was measured based on unenhanced and GA-enhanced images, obtained in the arterial, venous, transitional, as well as in the HBP. Measurements were performed by positioning nine separate circular regions of interest (ROIs), which were a minimum of 1 cm in diameter in each Couinaud liver segment including segments 4a and 4b. Regions of interest were selected avoiding visible vascular and biliary structures and the abdominal wall. RLE was calculated according to the following formula: RLE = (SI_post_ − SI_pre_)/SI_pre_, where SI_pre_ is unenhanced signal intensity and SI_post_ is signal intensity measured on GA-enhanced images in the HBP (16). SV was measured using syngo.via (Siemens, Erlangen, Germany) using the freehand volume of interest tool in the multimodal reading mode. Changes in RLE and SV are expressed as relative changes between baseline and follow-up MRI. Portosystemic collaterals were graded by their maximum axial diameter as absent, minor (<4 mm), or major (≥4 mm), respectively.

### 2.6. GA-MRI Response

GA-MRI response was defined by an increase in RLE which was paralleled by a decrease in SV, while patients with a decrease in RLE and an increase in SV were referred to as GA-MRI nonresponders.

### 2.7. Clinical Events during Follow-Up

Patients were followed for the development of (further) hepatic decompensation after IFN-free treatment. (Further) hepatic decompensation was defined by variceal (re)bleeding, incident ascites/worsening of ascites (requirement of paracentesis), and incident hepatic encephalopathy (HE)/worsening of HE (admission for grade 3/4 HE).

### 2.8. Statistical Analysis

Statistical analyses were performed using IBM SPSS Statistics 24 (IBM, Armonk, NY, USA) and GraphPad Prism 7 (GraphPad Software, La Jolla, CA, USA).

Categorical variables were presented as number (percentage) and scalable variables as median (interquartile range).

Mann–Whitney *U* and Kruskal–Wallis tests were used for group comparisons of continuous variables (i.e., comparison of treatment-induced changes in RLE and SV between patients with or without portosystemic collaterals). Intraindividual comparisons were performed using the Wilcoxon matched-pairs signed rank test (i.e., treatment-induced changes in SI, RLE, and SV). Spearman's correlation coefficient was calculated for correlation analyses (i.e., the correlation between the time interval from pretreatment MRI to treatment initiation and ΔRLE/ΔSV, the correlation between relative ΔRLE and ΔSV in treated patients, and the correlation between changes in platelet count/biochemical liver function tests and RLE/SV).

The development of (further) hepatic decompensation was analyzed using the Kaplan–Meier method; group comparisons were performed by the logrank test.

*P* values < 0.05 were considered as statistically significant. In case of multiple comparisons, *P* values were corrected by the Bonferroni procedure (i.e., comparison of pre- and posttreatment SI values on unenhanced and GA-enhanced images, obtained in the arterial, venous, transitional, as well as in the HBP).

## 3. Results

### 3.1. Patient and Treatment Characteristics

The majority of patients (*n*=19 of 31, 70%) had Child-Pugh (CP) A cirrhosis, with a median MELD of 9 (8–11) points. Fifty-two percent of patients (*n*=14) had esophageal varices (Table 1).

The median time between pretreatment MRI and treatment initiation was 11 (2–24) months. Posttreatment MRI was performed at a median of 10 (6–13) months after the end of treatment. The median time between pre- and posttreatment MRI was 20 (11–29) months. There was no correlation between the time interval from pretreatment MRI to treatment initiation and ΔRLE (*ρ*=−0.135; *P*=0.47) or ΔSV (*ρ*=0.063; *P*=0.736), or treatment duration and ΔRLE (*ρ*=0.324; *P*=0.075) or ΔSV (*ρ*=0.089; *P*=0.635). Similarly, there was no correlation between the time from end of treatment to posttreatment MRI and ΔRLE (*ρ*=0.03; *P*=0.873) or ΔSV (*ρ*=−0.062; *P*=0.739).

### 3.2. Changes in SI and RLE in Treated Patients

At both time points (pre- and post-IFN-free therapy), the liver showed the characteristic low SI on unenhanced scans with a gradual increase after GA administration, peaking in the HBP (Supplementary Figure 1).

In the HBP, SI posttreatment was statistically significantly increased, when compared to pretreatment: 447 (339–525) versus 551 (456–667); *P*=0.005. Furthermore, there was a statistically significant SI increase in the transitional phase (434 (343–500) versus 511 (408–597); *P*=0.02). The signal intensities in unenhanced scans (244 (219–270) versus 251 (220–278); *P*=1), arterial scans (330 (277–374) versus 338 (279–382); *P*=1), and venous images (460 (384–522) versus 496 (426–588); *P*=0.39) did not change statistically significantly from pre- to posttreatment (Supplementary Figure 1).

**Figure 2:**
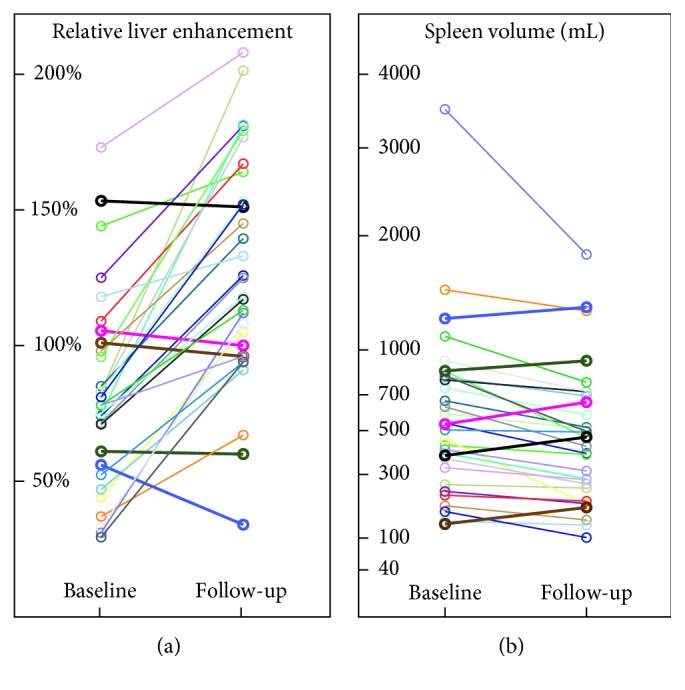
Changes in (a) relative liver enhancement (RLE) and (b) spleen volume before and after antiviral therapy. Patients who had a decrease in RLE were exactly the same patients who showed an increase in spleen volume.

**Figure 3:**
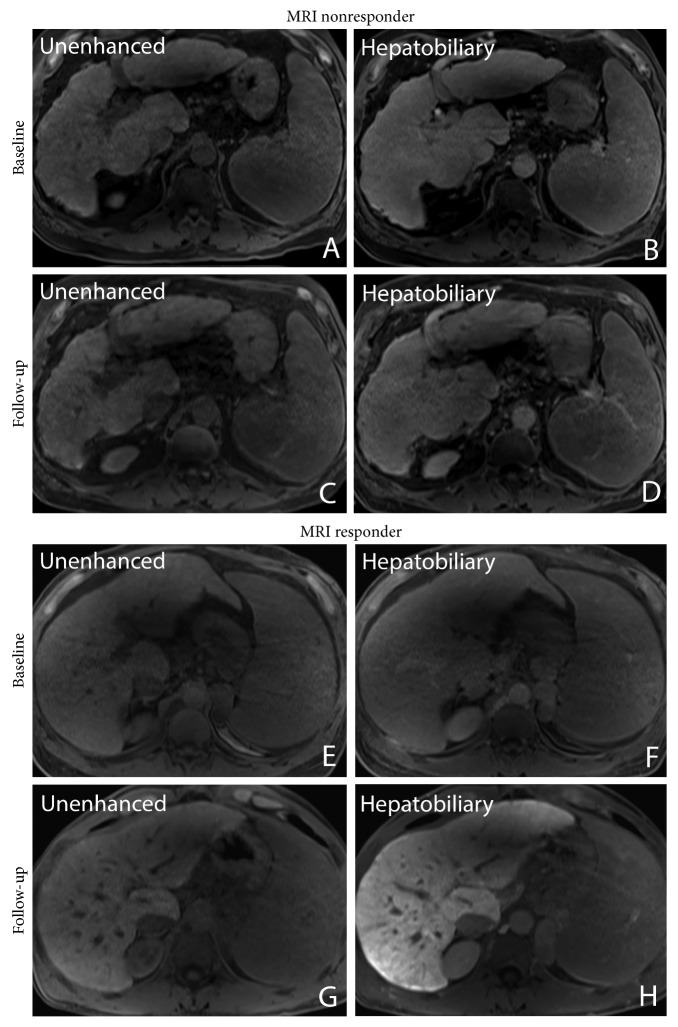
Pre- and posttherapy liver images unenhanced (A, C) and 20 minutes after gadoxetic acid administration in the hepatobiliary phase (B, D) in a 68-year-old male patient with a decrease in relative liver enhancement (RLE) from 56% to 34%. Note the portosystemic collaterals (graded as major portosystemic collaterals) indicating clinically significant portal hypertension. (E–F) Pre- and posttherapy images in a 50-year-old male patient without portosystemic collaterals who had an increase in RLE from 31% to 112%.

The RLE increased statistically significantly from pre- to posttreatment by 66% (20%–94%) in paired analysis (*P* < 0.001). Twenty-six patients (84%) showed an increase in RLE after antiviral therapy, whereas RLE decreased in 5 of 31 patients (Figures 2 and 3).

### 3.3. Changes in SV in Treated Patients

While RLE increased after IFN-free treatment, there was a statistically significant decrease in SV by −17% (−28% to −8%) in a paired analysis (*P* < 0.001). However, SV increased in 5 of 31 patients (16%), the identical patients who showed a decrease in RLE (Figure 2).

**Figure 4:**
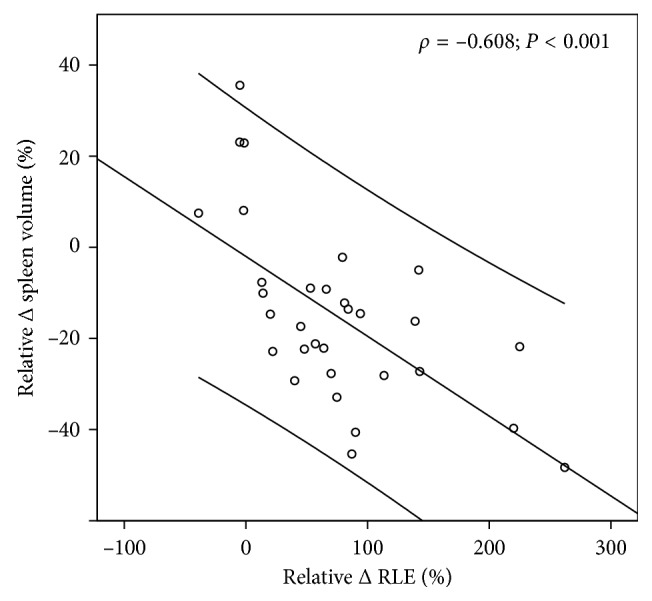
Correlation between the relative changes in relative liver enhancement (RLE) and spleen volume after antiviral therapy with interpolation line and 95% confidence interval.

We observed an inverse correlation of moderate strength between ΔRLE and ΔSV (*ρ*=−0.608; *P* < 0.001; Figure 4).

### 3.4. Changes in RLE and SV according to the Presence of Portosystemic Collaterals in Treated Patients

**Figure 5:**
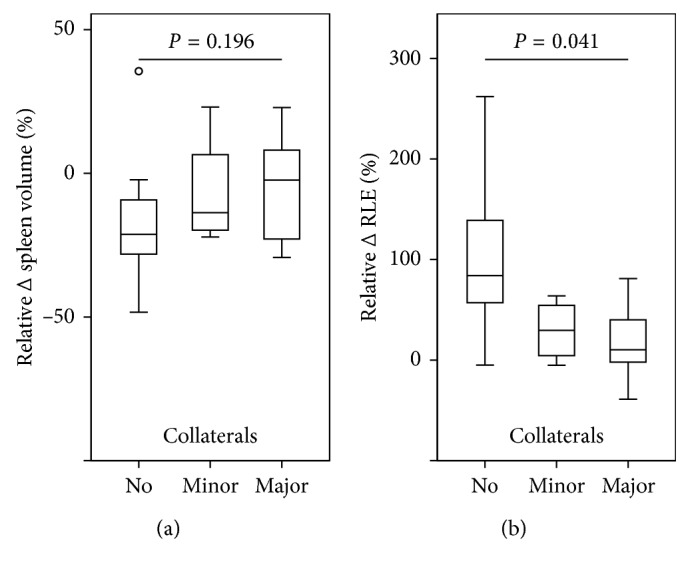
Relative changes in (a) spleen volume and (b) relative liver enhancement (RLE) after antiviral therapy according to the presence of portosystemic collaterals.

The increase in RLE was significantly lower in patients with portosystemic collaterals indicating the presence of clinically significant portal hypertension. We observed an increase in the RLE after antiviral therapy by 83% (53% to 139%), 45% (−5% to –70%), 10% (−2% to –81%) in patients without minor, with minor, and major portosystemic collaterals, respectively (*P*=0.041; Supplementary Figure 2). Relative decreases in spleen volume among patients were as follows: no portosystemic collaterals: −22% (−29% to −9%), followed by patients with minor portosystemic collaterals: −14% (−21% to 23%), and major portosystemic collaterals: −2% (−23%–8%) (Figure 5). The observed differences did not attain statistical significance (*P*=0.196).

There were no statistically significant correlations between changes in platelet count/biochemical liver function tests and RLE/SV (Supplementary Table 1 and Supplementary Figure 2).

### 3.5. Changes in RLE and SV in the Untreated Group

The median time between initial and follow-up MRI in the untreated cohort was 11 (2–24) months. In contrast to treated patients, we observed a decrease in RLE by −11% (−25% to −3%; *P*=0.019) and an increase in SV by 23% (7%–43%; *P*=0.004; Supplementary Figure 3).

### 3.6. Development of (Further) Hepatic Decompensation according to GA-MRI Response to IFN-Free Treatment

During a median posttreatment follow-up of 25.2 (17.9–34.8) months, 6 patients developed (further) hepatic decompensation, with variceal bleeding (*n*=1), ascites (*n*=2), or HE (*n*=3) being the first events. Two patients underwent liver transplantation, and one patient died after developing further hepatic decompensation.

**Figure 6:**
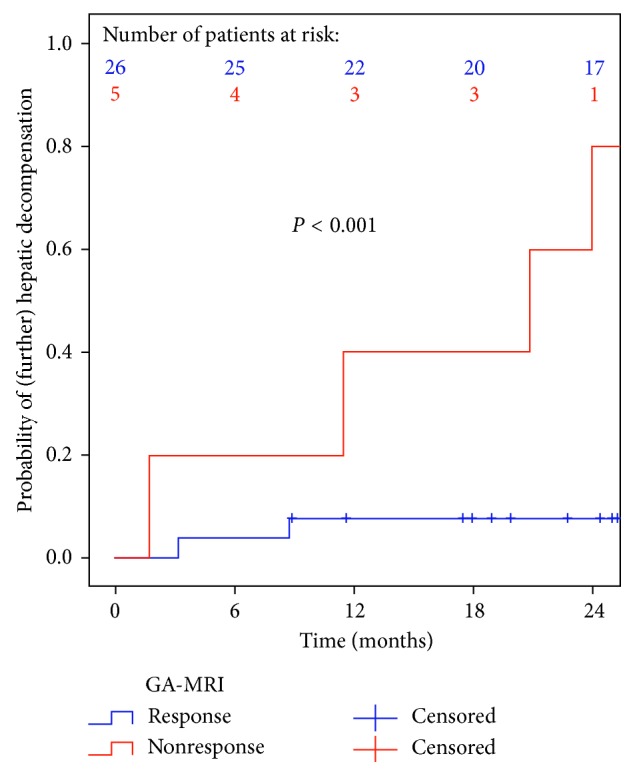
Development of (further) hepatic decompensation according to gadoxetic acid-enhanced MRI (GA-MRI) response to interferon-free treatment.

Interestingly, GA-MRI-nonresponse was associated with a substantially increased risk of (further) hepatic decompensation 2 years after the end of treatment: 80% versus 8%; *P* < 0.001 (Figure 6).

## 4. Discussion

The results of our study demonstrate that GA-MRI might be able to distinguish between low and high risk individuals for (further) hepatic decompensation (GA-MRI-nonresponse) after HCV eradication. Thus, GA-MRI may identify patients with improving liver function (i.e., regression of liver fibrosis and portal hypertension) and those with persistent liver damage despite SVR to IFN-free therapies.

Overall, we observed a statistically significant negative correlation of moderate strength between the relative change in RLE, possibly indicative of hepatic inflammation and fibrosis [16] and spleen volume, potentially indicative of portal hypertension [17, 18]. The majority (84%) of patients showed an increase in RLE after HCV eradication, which was accompanied by a decrease in SV [17, 18]. In contrast, untreated controls showed contrary changes in RLE and SV, which is in line with progressive liver disease due to CHC.

If confirmed by further studies evaluating liver histology and/or hepatic venous pressure gradient (HVPG), GA-MRI may serve as a clinically useful biomarker for monitoring the improvement in liver function associated with regression of hepatic inflammation and possibly with regression of HCV-induced hepatic fibrosis and portal hypertension after successful HCV eradication with IFN-free regimens. It is well known that liver fibrosis, and consequently, portal hypertension drives the development of liver-related events in patients with CHC. Therefore, it is essential to reassess both variables after HCV eradication.

It is important to consider that clinically significant portal hypertension may persist despite normalization of liver function tests after HCV eradication, and patients with clinically significant portal hypertension remain at considerable risk for hepatic decompensation, even after achieving SVR [19, 20]. Likewise, advanced liver fibrosis may persist, and thus, patients also remain at significant risk for HCC development. Regression of liver fibrosis and portal hypertension can be evaluated with liver biopsy and HVPG measurement, respectively [21, 22]. Since a decrease in HVPG translates into a clinically meaningful benefit, it is considered an acceptable surrogate endpoint for etiologic therapies [23]. However, the use of liver biopsy and HVPG measurement for monitoring of patients after HCV eradication is limited by its invasiveness. Thus, the development of noninvasive methods to distinguish between patients who benefited from IFN-free therapy (i.e., resolved liver fibrosis and portal hypertension) and patients who remain at a considerable risk for liver-related events is of high clinical relevance.

In the current study, RLE significantly increased from pre- to posttreatment in paired analysis. The observed dynamics of SV support these results. Our results are in line with that of other clinical studies reporting that there is a substantial variation in the regression of liver fibrosis and portal hypertension after HCV eradication [21, 22, 24].

In a study [21] comprising 60 patients who underwent HVPG measurement before and after antiviral therapy, the authors observed very homogenous decreases in HVPG in patients with subclinical portal hypertension (HVPG 5–9 mmHg). However, in patients with clinically significant portal hypertension (CSPH; HVPG ≥ 10 mmHg), changes in HVPG were heterogeneous, and some patients even had an increase in HVPG despite an SVR. These findings are again in line with the results of the present study, since portosystemic collaterals only occur in patients with CSPH [23], and patients with portosystemic collaterals showed lower increases in RLE and a trend toward a less pronounced decrease in SV.

Another recently published study evaluated transient elastography (TE), the most commonly used noninvasive method for the staging of liver fibrosis and for monitoring liver disease regression after SVR, which was assessed by paired HVPG measurements [24]. Interestingly, the diagnostic performance of TE was suboptimal in patients with more advanced liver disease. This emphasizes the need for other noninvasive markers for risk stratification, for example, GA-MRI [25].

Importantly, GA-MRI predicted the development of (further) hepatic decompensation, which is a highly relevant direct endpoint in patients with advanced chronic liver disease [23]. Although the significance of this finding is limited by the low number of events, GA-MRI nonresponders showed a substantially worse prognosis, with an 80% probability of having developed (further) hepatic decompensation 2 years after the end of treatment. In contrast, GA-MRI responders had a more favorable prognosis.

Some limitations should be considered when interpreting the results of our study: firstly, due to the retrospective study design, we cannot exclude a selection bias. We did not obtain liver biopsies or HVPG pre- and posttreatment; however, the changes in RLE (surrogate of hepatic inflammation and fibrosis) correlated well with the changes in splenic volume (surrogate of portal hypertension). Moreover, the results obtained in the untreated cohort (showing decreases in RLE and increases in SV, which is in line with the natural history of CHC) support the sensitivity of GA-MRI for dynamic changes in chronic liver disease. Of note, the time interval between the GA-MRI assessments differed between treated and untreated patients. Thus, we abstained from direct comparisons between both groups. Secondly, GA-MRI response to IFN-free treatments also predicted clinical events. Sample size was rather small; however, it was sufficient to attain statistically significant results. Lastly, the median time between pretreatment MRI and treatment initiation was rather long and showed significant variability, 11 (2–24) months; however, there was no correlation between this time interval and changes in RLE or SV.

## 5. Conclusions

This is the first study demonstrating that GA-MRI might be able to distinguish between individuals at low and high risk of (further) hepatic decompensation (GA-MRI-nonresponse) after HCV eradication. Thus, it might have important prognostic implications. If confirmed by larger prospective studies, GA-MRI might facilitate individualized post-SVR surveillance.

## Figures and Tables

**Table 1:** Patient characteristics at the time of pretreatment MRI as well as treatment characteristics.

Age (years)	61 (53–66)
Age (female) (years)	59 (56–68)
Age (male) (years)	61 (52–63)

Sex	
Male	24 (77%)
Female	7 (23%)

HCV genotype	
1	24 (77%)
3	2 (7%)
4	5 (16%)

Cirrhosis	27 (87%)
CP stage A	19 (70%)^*∗*^
CP stage B	8 (30%)^*∗*^
MELD points	9 (8–11)^*∗*^
History of variceal bleeding	0 (0%)^*∗*^
Varices	14 (52%)^*∗*^
Small	10 (37%)^*∗*^
Large	4 (15%)^*∗*^

Platelet count (G × L^−1^)	104 (77–146)

Albumin (g × L^−1^)	39.6 (35.7–41.8)

Bilirubin (mg × dL^−1^)	0.92 (0.64–1.48)

Prothrombin time (%)	77 (64.3–86.3)

Treatment-experienced	22 (71%)

Treatment regimen	
SOF/SMV	6 (19%)
SOF/DCV	19 (61%)
SOF/LDV	5 (16%)

Treatment duration	
12 weeks	10 (32%)
16 weeks	5 (16%)
20 weeks	2 (6%)
24 weeks	14 (45%)

^*∗*^Referring only to patients with cirrhosis. HCV: hepatitis C virus; CP: Child-Pugh score; MELD: model for end-stage liver disease; SOF: sofosbuvir; SMV: simeprevir; DCV: daclatasvir; LDV: ledipasvir.

**Table 2:** Imaging parameters.

MRI unit	3.0 Tesla, TrioTrim, Siemens, Erlangen, Germany

Coil	Combined six-element phased-array abdominal coil and fixed spine coil

Axial, three-dimensional breath-hold, T1-weighted, gradient-echo sequences (T1-3D GRE), i.e., VIBE	FOV^*∗*^: 350–400 × 350 mm
	FS: SPAIR
	AF: 2
	Sequence duration^*∗*^: 18–21 s
	Section thickness^*∗*^: 1.7 mm; gap: 0 mm
	TR^*∗*^: 2.67 ms; TE: 0.97 ms
	FA: 13°

Contrast medium	i.v. bolus injection of 0.025 mmol/kg body weight of gadoxetic acid at 1 mL/s and 20 mL saline flush

Imaging time points	Unenhanced
	AP (immediately)
	PVP (70 s)
	TP (5 min)
	HBP (20 min)

T1-weighted axial in-phase	TR^*∗*^: 130; TE: 2.46
	FA: 70°
	FOV: 640 × 500

T1-weighted axial opposed-phase	TR^*∗*^: 131; TE: 3.69
	FA: 70°
	FOV^*∗*^: 320 × 250

T2 HASTE	TR^*∗*^: 1600; TE: 100
	FA: 150°
	FOV^*∗*^: 512 × 448

DWI	B 50–600 and ADC map
	TR^*∗*^: 4404, TE: 73
	FA: 90°
	VOF^*∗*^: 384 × 288

^*∗*^Individual adjustment depending on patient size and breath hold capability. FOV: field of view; FS: fat sat; SPAIR: spectral adiabatic inversion-recovery technique; AF: acceleration factor; TR: repetition time; TE: echo time; FA: flip angle (anteroposterior phase direction); GRE: gradient-recalled echo; VIBE: volumetric interpolated breath-hold examination; HBP: hepatobiliary phase; AP: arterial phase; PVP: portal venous phase; TP: transitional phase; HBP: hepatobiliary phase; HASTE: half-fourier acquisition single-shot turbo spin-echo; DWI: diffusion-weighted images; ADC: apparent diffusion coefficient.

## Supplementary Materials

*Supplementary 1*.
Figure 1: gadoxetic acid-enhanced liver MRI at both time points (pre- and posttherapy) showed the characteristic of low signal intensity in unenhanced scans with a gradual increase over time to a peak in the hepatobiliary phase (HBP) 20 minutes after administration of gadoxetic acid. A statistically significant difference between pre- (baseline) and posttherapy (follow-up) signal intensity was observed in the transitional phase (TP) and the HBP. All *P* values are Bonferroni adjusted.

*Supplementary 2*.
Figure 2: changes in (A) platelet count and biochemical liver function tests, ((B) albumin, (C) bilirubin, and (D) prothrombin time) after antiviral therapy.

*Supplementary 3*.
Figure 3: comparison of relative changes in (A) relative liver enhancement (RLE) and (B) spleen volume between an untreated control group and treated patients.

*Supplementary 4*.
Table 1: association between relative changes in laboratory liver function tests and relative liver enhancement (RLE)/spleen volume (SV).
