# Supplementary material for: Noninvasive Monitoring of Liver Disease Regression after Hepatitis C Eradication Using Gadoxetic Acid-Enhanced MRI
Source: Contrast Media Mol Imaging. 2018 Jul 12;2018:8489709. doi: 10.1155/2018/8489709 (PMC6079600; doi:10.1155/2018/8489709)
Supplement: Supplementary 4. — Table 1: association between relative changes in laboratory liver function tests and relative liver enhancement (RLE)/spleen volume (SV). [file 8489709.f4.docx]

**Supplementary Table 1**

|  | Relative Δ RLE, % | | Relative Δ SV, % | |
| --- | --- | --- | --- | --- |
|  | ρ | P value | ρ | *P* value |
| Relative Δ platelet count | 0.035 | 0.851 | -0.201 | 0.278 |
| Relative Δ albumin | 0.248 | 0.179 | -0.299 | 0.103 |
| Relative Δ bilirubin | -0.237 | 0.199 | 0.084 | 0.652 |
| Relative Δ prothrombin time | -0.02 | 0.916 | 0.014 | 0.942 |
